# NUTRITIONAL STATUS IN CHILDREN WITH CANCER: COMPARISON OF DEUTERIUM OXIDE DILUTION WITH BIOELECTRIC IMPEDANCE ANALYSIS AND ANTHROPOMETRY

**DOI:** 10.1590/1984-0462/2021/39/2019209

**Published:** 2020-08-03

**Authors:** Estela Beatriz Behling, José Simon Camelo, Eduardo Ferriolli, Karina Pfrimer, Jacqueline Pontes Monteiro

**Affiliations:** a Universidade Federal do Rio Grande do Sul, Porto Alegre, RS, Brazil.; b Universidade de São Paulo, Ribeirão Preto, SP, Brazil.

**Keywords:** Body composition, Cancer, Child, Anthropometry, Electric impedance, Deuterium oxide, Composição corporal, Câncer, Criança, Antropometria, Impedância bioelétrica, Óxido de deutério

## Abstract

**Objective:**

To explore changes in the nutritional status of pediatric cancer patients before and after chemotherapy and evaluate the correlation between deuterium oxide dilution, bioelectric impedance analysis, and anthropometry for assessment of body composition.

**Methods:**

This study included 14 children (aged 5.6 to 13.6 years) and classified them as having hematologic or solid tumors. They had their body composition analyzed according to deuterium oxide, bioelectric impedance, and anthropometric measurements before the first chemotherapy cycle and after three and six months of therapy.

**Results:**

The patients in the hematologic tumor group had an increase in weight, height, body mass index, waist, hip, and arm circumference, subscapular skinfold thickness, and fat mass with the isotope dilution technique during chemotherapy. In the solid tumor group, the children showed a reduction in fat-free mass when assessed by bioimpedance analysis. We found a positive correlation between the triceps skinfold thickness and fat mass determined by bioimpedance analysis and deuterium oxide. The arm muscle circumference correlated with the fat-free mass estimated by bioimpedance analysis and deuterium oxide.

**Conclusions:**

Patients with hematologic tumors had an increase in body weight, height, and fat mass, which was not identified in the solid tumor group. The positive correlation between anthropometry (triceps skinfold thickness and arm muscle circumference), deuterium oxide dilution, and bioelectric impedance analysis shows the applicability of anthropometry in clinical practice.

## RESUMO

**Objetivo:**

Verificar mudanças no estado nutricional de crianças com câncer antes e após o tratamento quimioterápico e avaliar a correlação entre diluição de óxido de deutério, análise de impedância bioelétrica (BIA) e dados antropométricos.

**Métodos:**

Quatorze crianças (entre 5,6 e 13,6 anos de idade) foram incluídas e classificadas como tendo tumores hematológicos ou sólidos. A composição corporal foi medida pelo óxido de deutério, impedância bioelétrica e medidas antropométricas antes da primeira quimioterapia e após três e seis meses de terapia.

**Resultados:**

Os pacientes do grupo de tumores hematológicos aumentaram o peso, a estatura, o índice de massa corporal, a circunferência da cintura, quadril e braço, dobra cutânea subescapular e a massa gorda com a técnica de diluição isotópica durante o tratamento quimioterápico. No grupo de tumores sólidos, as crianças mostraram uma redução na massa magra quando avaliadas por análise de impedância. Houve uma correlação positiva entre a dobra cutânea tricipital e a massa gorda determinadas pela análise de impedância e pelo óxido de deutério. A circunferência muscular do braço correlacionou-se com a massa magra estimada pela análise de impedância e pelo óxido de deutério.

**Conclusões:**

Pacientes com tumores hematológicos tiveram aumento no peso corporal, estatura e massa gorda, o que não foi observado naqueles com tumores sólidos. A boa correlação entre a antropometria (dobra cutânea tricipital e circunferência muscular do braço), a diluição do óxido de deutério e a análise da impedância bioelétrica mostra a aplicabilidade da antropometria na prática clínica.

## INTRODUCTION

Changes in nutritional status are frequent during the first months of treatment in children with cancer. Nutritional status is associated with tolerance to chemotherapy, number and intensity of infections, and cancer prognosis in the pediatric population. Nonetheless, conventional anthropometric measurements cannot identify the changes related to fat mass (FM) and fat-free mass (FFM) during the first months after diagnosis.^1^ ^,^ ^2^

Historically, the nutritional status of cancer patients has been evaluated by various objective measures, including anthropometric (weight, arm muscle circumference, and triceps skinfold thickness) and biochemical (serum albumin, transferrin assays, and nitrogen balance studies) ones.^3^ ^,^ ^4^

Bioelectrical impedance analysis (BIA) became popular in the past decade due to its practical advantages as a non-invasive, safe, inexpensive, and portable method for assessing body composition. BIA has been validated for the assessment of body composition and nutritional status in various populations, including cancer patients.^5^

BIA measures tissue conductivity and can assess total body water (TBW). In the absence of edema or ascites, TBW may be used to monitor the significance of weight change. It is an easy, reliable, and portable technique for measuring body composition that is applicable for fieldwork and less expensive than the deuterium oxide (D_2_O) method.^6^ ^,^ ^7^

The D_2_O stable isotope dilution is a reference technique for measuring total body water (TBW). After the ingestion and equilibration of a known dose of D_2_O in the water compartments of the body, D_2_O concentration acts as a marker for TBW from which FFM and FM are derived. The D_2_O dilution technique assumes a constancy of the hydration fraction of FFM, which varies between individuals and during growth and development. This technique is a safe and well-established standard method for assessing body water compartments even during childhood.^8^

The gold standard for measuring TBW is isotope dilution, but this method is only appropriate in research settings.^8^ ^,^ ^9^ Estimates of TBW in children derived from measurements made by isotope dilution have been proposed, but the technique is very expensive and not practical. As a result, BIA and anthropometric measurements have been used in clinical practice.^10^

This study aimed to explore changes in the nutritional status of pediatric cancer patients before and after chemotherapy and evaluate the correlation between deuterium oxide dilution, bioelectric impedance analysis, and anthropometry for assessment of body composition.

## METHOD

All children with cancer aged 5-15 years, treated at this hospital from August 1, 2006 to November 30, 2007 were considered eligible to participate in the study. Fourteen participants agreed to participate by signing the informed consent form. Children who had previously been treated for cancer, Down syndrome or those who were not able to be assessed by anthropometric measures were excluded.

Fourteen children (seven boys and seven girls) with cancer were enrolled in the study and divided into two groups: Hematologic Tumor Group (n=7) and Solid Tumor Group (n=7). The mean age at the beginning of the study was 10.13 years (range 5.6-13.6). They were evaluated according to dietary intake, anthropometry, deuterium dilution, and BIA techniques at baseline (M0) and after 3 (M1) and 6 (M2) months of chemotherapy.

Data were collected at a reference hospital. The same investigator took all measurements at a similar time to avoid any systematic technical bias. During the wait between the administration of deuterium oxide and the collection of the final saliva sample, BIA and anthropometric measures were performed. Consequently, the hydration status was likely to have remained unchanged throughout the measurement period.

A trained dietitian took anthropometric measures by using standardized techniques. Standing stretched height (0.1 cm) was measured with a wood stadiometer. Body weight (0.01 kg) was measured with digital scales while subjects wore light clothing. Body mass index (BMI) was calculated as weight/height^2^ (kg/m^2^). Skinfold thickness was measured in triplicate to the nearest 0.2 mm at three sites (biceps, triceps, and subscapular) using calipers.^11^ Mid upper arm circumference (MUAC) was measured in the mid-point of the olecranon process of the ulna and the acromial process of the scapula at a right angle. Waist circumference was taken in the standing position at the umbilicus. Hip circumference was measured in the standing position around the widest part of the hips and buttocks. All skinfold measurements were performed in the same order on the left side of the body, which is usually the non-dominant one, including MUAC. Anthropometric measurements corresponded to the mean of triplicates. The same researcher took all measurements.

BIA was performed using a bioelectrical impedance analyzer (model Biodynamics BIA 450, Biodynamics Corp, USA)^®^. BIA was conducted while patients were lying supine on a bed or examination table, with their arms and legs apart and not touching the torso; the measures were performed after the subjects had remained in the supine position for ≥10 min so their body fluids could reach equilibrium. The skin was cleaned with alcohol before the electrodes were placed, and all metal objects were removed from the participants before the measures were taken. All evaluations were conducted on the patient’s right side using the four surface standard electrode (tetrapolar) technique on the hand and foot.^12^ One electrode was placed on an imaginary line on the protruding bone on the little finger side of the wrist to bisect the ulnar head of the right hand. Another electrode was placed below the knuckle of the middle finger on the right hand. A third electrode was placed on an imaginary line on the protruding bone on the big toe side of the ankle to bisect the medial malleolus of the right foot. The fourth and final electrode was placed just below the middle toe of the right foot, which is the standard procedure for body impedance measures.

The calibration of the instrument was checked daily with the use of standard resistors purchased with the analyzer. The same person performed all BIA measurements.

After an eight-hour fast, subjects were given D_2_O dilution. The participants first provided a pre-dose fasting saliva sample to determine the natural deuterium content. Next, an accurately weighed dose equivalent to 0.07 g/kg body weight of D_2_O (D_2_O; 99.8% purity, Cambridge Isotope Laboratories Inc, Andover, MA) was administered orally to each subject, and a sample of the dose was kept for analysis. Saliva samples (~2 mL directly in small sterile vials) were collected immediately before D_2_O administration, and again, two and three hours after the D_2_O dose.

Enrichment of saliva samples was determined by Mass Spectrometry (Europe Scientific Hydra System, Cheshire, United Kingdom) after twelve hours in equilibrium with 100% hydrogen in platinum catalyzed in aluminum. Children seem to have a higher aqueous fraction of FFM than young adults;^13^ therefore, we calculated FFM assuming that the hydration fraction of FFM depends on the age and gender of the child as described by Lohman,^14^ and the values found ranged between 73.8 and 79%. Weight was measured in a fasting state in the early morning for all calculations, and FM was determined as the difference between FFM and body weight. All saliva samples were stored at -20°C and sent to the Mass Spectrometry Laboratory for analysis.

The purpose of the study and a description of the testing protocol were explained to each subject and their parents. A parent of each participant signed the informed consent form, and the study protocols were approved by and followed the requirements of the Ethics Committee (HCRP/USP 15411/2005).

Analyses were performed using *Statistical Package for the Social Sciences* (SPSS), version 18.0 (SPSS Inc., Chicago, IL, USA). Initially, the continuous variables were tested for normality. Values were expressed as median (minimum-maximum) because most variables had abnormal distribution. We used the Friedman t-test. Differences between proportions were calculated using the chi-square test. We calculated correlations with Spearman’s rank correlation coefficient. A difference was considered statistically significant if p<0.05.

## RESULTS

Twenty-one subjects agreed to participate in the study. The final study sample included 14 children with cancer, seven females and seven males, with ages ranging from 5.6 to 13.6 years. Seven subjects were excluded due to incomplete data (no cancer, no chemotherapy, died, treatment in another institution). Table 1 presents a description of the sample of 21 individuals according to age, gender, and diagnosis.

**Table 1:** Clinical characteristics and diagnosis of the patients at baseline.

Patient number	Gender	Age (years)	Diagnosis
1	M	11	ALL
2	M	13	AML
3	F	10	ALL
4	F	5	-------*
5	F	9	--------*
6	M	10	Hodgkin’s lymphoma
7	F	13	Synovial sarcoma*
8	F	8	Rhabdomyosarcoma
9	F	10	Non-Hodgkin’s lymphoma
10	F	6	Metastatic medulloblastoma
11	F	6	Brainstem malignancy*
12	M	10	AML + Fanconi anemia
13	M	5	Spinal tumor
14	F	13	Embryonal rhabdomyosarcoma
15	M	11	Oligoastrocytoma
16	F	12	-------*
17	F	13	Central nervous system germinoma
18	F	6	Ewing sarcoma
19	M	10	AML
20	F	15	------*
21	M	6	Central nervous system germinoma*

F: female; M: male; ALL: acute lymphoblastic leukemia; AML: acute myeloid leukemia; *patients excluded from the study [no chemotherapy (n=7); died during treatment (n=11), continued treatment in another institution (n=21); did not have a confirmed diagnosis of cancer (n=4, 5, 16, 20)].

Table 2 shows the anthropometric characteristics of the children. Patients from the Hematologic Tumor Group had an increase in the weight, height, BMI, waist, hip, and arm circumferences, and subscapular skinfold thickness.

**Table 2:** Anthropometric measures of children at each time point.

	First evaluation (M0)	Second evaluation (M1)	Third evaluation (M2)	p-value
Hematologic Tumor Group (n=7)				
Age (y)	10.2 (9.9-13.4)	10.4 (10.2-13.7)	10.7 (10.4-13.9)	
Height (cm)	150.0 (118.0-162.0)	150.0 (118.5-164.0)	150.5 (119.0-164.0)	0.074
Weight (kg)	36.3 (17.9-45.7)	39.6 (18.0-52.1)	41.7 (19.4-61.1)	0.004^*^
BMI (kg/cm^2^)	16.4 (12.7-19.3)	17.1 (12.8-22.0)	19.8 (13.7-22.7)	0.005*
Waist circumference (cm)	61.0 (52.0-76.0)	69.0 (53.0-81.0)	72.0 (50.0-82.0)	0.018*
Hip circumference (cm)	74.0 (53.5-84.0)	75.0 (54.0-93.0)	81.0 (54.0-101.0)	0.004^*^
MUAC (cm)	20.0 (13.0-22.5)	21.0 (13.5-25.0)	21.5 (15.0-27.5)	0.003*
MUAMC (cm)	16.8 (12.1-17.9)	17.2 (12.6-20.0)	18.2 (14.1-20.0)	0.028*
Skinfold thickness				
Triceps (mm)	7.0 (3.0-18.0)	15.0 (3.0-18.0)	14.0 (3.0-25.0)	0.047*
Biceps (mm)	6.0 (2.0-14.0)	7.0 (3.0-14.0)	10.0 (3.0-16.0)	0.044*
Subscapular (mm)	6.0 (2.0-14.0)	7.0 (3.0-16.0)	10.0 (5.0-16.0)	0.012^*^
Solid Tumor Group (n=7)				
Age (y)	8.5 (5.6-13.6)	8.8 (5.8-13.8)	9.0 (6.2-14.1)	
Height (cm)	128.0 (111.0-161.0)	128.5 (111.0-163.0)	129.5 (111.0-163.0)	0.006*
Weight (kg)	25.8 (17.1-41.3)	32.6 (18.4-47.0)	30.3 (19.0-42.9)	0.062
BMI (kg/cm^2^)	15.3 (13.9-18.8)	15.3 (14.5-23.8)	15.2 (14.8-21.0)	0.459
Waist circumference (cm)	61.0 (50.0-78.0)	72.0 (53.0-79.0)	65.0 (54.0-71.0)	0.121
Hip circumference (cm)	69.0 (55.0-82.0)	79.0 (56.0-86.0)	75.0 (58.0-83.0)	0.241
MUAC (cm)	18.5 (16.0-23.0)	19.0 (15.0-24.0)	19.5 (16.0-23.0)	0.630
MUAMC (cm)	16.6 (14.1-18.0)	16.8 (13.4-17.7)	16.7 (14.1-18.2)	0.772
Skinfold thickness				
Triceps (mm)	10.0 (6.0-16.0)	9.0 (5.0-20.0)	11.0 (6.0-20.0)	0.607
Biceps (mm)	8.0 (4.0-9.0)	7.0 (4.0-14.0)	7.0 (4.0-14.0)	0.453
Subscapular (mm)	7.0 (3.0-12.0)	8.0 (3.0-18.0)	10.0 (4.0-15.0)	0.056

Data are expressed as median values and ranges (minimum, maximum); BMI: body mass index; cm: centimeter; mm: millimeter; kg: kilogram; MUAC: mid-upper arm circumference; MUAMC: mid-upper arm muscle circumference. *p<0.05. Friedman test.

We found a strong correlation between triceps skinfold thickness and FM using BIA (r=0.534; p=0.049/ r=0.734; p=0.007/ r=0.851; p=0.000; at M0, M1, and M2, respectively) and D_2_O (r=0.694; p=0.006/ r=0.820; p=0.000/ r=0.813; p=0.001; at M0, M1, and M2, respectively), according to Spearman’s correlation.

MUAMC correlated with FFM in kg estimated by BIA (r=0.841; p=0.000/ r=0.797; p=0.001/ r=0.863; p=0.000; at M0, M1, and M2, respectively) and D_2_O (r=0.474; p=0.087/ r=0.770; p=0.001/ r=0.793; p=0.001; at M0, M1, and M2, respectively) using Spearman’s correlation.

FFM (kg) estimated by BIA presented no significant difference in the three moments. D_2_O showed a decrease in FM (p=0.011) and an increase in FFM (kg and %) (p=0.042 and p=0.011) during the treatment in the Hematologic Tumor Group (Table 3).

**Table 3:** Body composition of children at each time point.

	First evaluation (M0)	Second evaluation (M1)	Third evaluation (M2)	p-value
Hematologic Tumor Group (n=7)				
TBW_BIA_ (L)	19.6 (11.0-28.6)	23.0 (12.4-28.4)	21.2 (11.7-24.6)	0.311
TBW_D2O_ (%)	56.4 (50.5-72.2)	58.5 (53.1-63.7)	50.3 (45.1-54.4)	0.009*
TBW_BIA_ (%)	74.9 (70.3-77.1)	74.6 (71.2-77.5)	73.4 (61.8-74.8)	0.069
FFM_D2O_ (kg)	27.9 (2.0-39.6)	33.2 (25.0-39.4)	31.3 (23.8-36.0)	0.042*
FFM_BIA_ (kg)	26.9 (14.6-37.1)	30.9 (16.0-38.8)	31.5 (15.6-34.6)	0.115
FFM_D2O_ (%)	75.1 (66.3-93.7)	74.5 (69.7-84.5)	66.7 (58.8-72.3)	0.011*
FFM_BIA_ (%)	80.2 (70.4-87.7)	80.5 (72.0-88.9)	71.4 (53.2-80.4)	0.069
FM_D2O_ (kg)	8.8 (2.7-15.4)	10.1 (5.6-15.8)	16.3 (11.3-25.2)	0.011*
FM_BIA_ (kg)	5.2 (3.3-8.7)	6.0 (2.0-12.9)	8.5 (3.8-28.6)	0.074
FM_D2O_ (%)	24.9 (6.3-33.7)	25.6 (15.5-30.3)	33.3 (27.7-41.2)	0.011*
FM_BIA_ (%)	19.8 (12.3-29.3)	19.5 (11.1-28.0)	25.0 (19.6-46.1)	0.069
Solid Tumor Group (n=7)				
TBW_BIA_ (L)	20.0 (9.7-23.4)	16.1 (13.1-25.8)	15.9 (13.1-24.3)	0.152
TBW_D2O_ (%)	62.5 (52.2-67.9)	57.2 (39.8-66.8)	53.9 (45.2-66.2)	0.565
TBW_BIA_ (%)	76.2 (70.2-88.0)	72.9 (68.8-80.5)	73.7 (68.6-79.6)	0.867
FFM_D2O_ (kg)	21.5 (14.1-31.2)	20.0 (15.8-32.1)	21.3 (16.3-30.6)	0.867
FFM_BIA_ (kg)	25.0 (12.6-31.8)	20.9 (16.3-35.4)	20.7 (5.6-33.0)	0.034*
FFM_D2O_ (%)	80.1 (71.4-87.5)	75.7 (51.7-85.7)	71.4 (59.8-86.0)	0.368
FFM_BIA_ (%)	77.0 (69.9-96.9)	75.3 (60.7-89.2)	71.0 (11.7-86.8)	0.368
FM_D2O_ (kg)	5.1 (3.0-11.8)	9.8 (2.6-15.8)	11.3 (2.7-16.9)	0.368
FM_BIA_ (kg)	4.6 (0.8-12.3)	11.4 (2.1-13.7)	9.9 (2.5-14.3)	0.495
FM_D2O_ (%)	19.9 (12.5-28.7)	24.3 (14.3-48.3)	28.7 (14.0-40.0)	0.368
FM_BIA_ (%)	23.0 (3.1-30.2)	24.7 (10.9-39.3)	29.0 (13.2-88.3)	0.368

Data are expressed as median values and rages (minimum, maximum); BIA: bioimpedance analysis; D_2_O: deuterium oxide; FFM: fat-free mass; FM: fat mass; kg: kilogram; L: liter; TBW: total body water. *p<0.05. Friedman test.

Spearman’s correlation is strong between BIA and D_2_O for: FFM (kg) (r=0.955; p≤0.001/ r=0.981; p≤0.001/ r=0.902; p≤0.001; at M0, M1, and M2, respectively), FM (kg) (r=0.833; p≤0.001/ r=0.929; p≤0.001/ r=0.940; p≤0.001; at M0, M1, and M2, respectively), and TBW (%) (r=0.514; p≤0.001/ r=0.826; p≤0.001/ r=0.836; p≤0.001; at M0, M1, and M2, respectively) (Table 3).

## DISCUSSION

There is a growing need for accurate body composition measurements in children with cancer. Many different techniques are available, from relatively easy-to-use, cheap methods, including weight, height, BMI, circumferences, and skinfold measurement, to more expensive and technical approaches, such as BIA, D_2_O, dual-energy X-ray absorptiometry (DXA), and others.^15^ ^,^ ^16^ ^,^ ^17^ ^,^ ^18^ Each of these methods has advantages and limitations. The current study aimed to determine the association between different body composition measures (anthropometry, BIA, and D_2_O dilution) to find a useful, quick, and easy instrument for daily practice.

We have shown that BIA provides a reliable estimation of body composition in children with cancer. Results obtained with the BIA method correlated well with those obtained with D_2_O. We found no significant difference between methods in the measurement of FFM or FM. Although the TBW (%) estimated with BIA was higher than that obtained with the D_2_O method in both groups, the difference was fairly small. It is difficult to ascertain which of the methods has the correct estimation in this field setting. Numerous studies have revealed that BIA is a sensitive method to describe body composition in children with different pathologies, including cancer, especially when they do not have edema.^4^ ^,^ ^6^ ^,^ ^7^ ^,^ ^19^ ^,^ ^20^ ^,^ ^21^ ^,^ ^22^

Solid tumors have a negative impact on the nutritional status of children, which is not always identified in hematologic cases.^3^ ^,^ ^18^ FFM is depleted in solid tumors, and food intake seems not to be sufficient to avoid this event.^23^ ^,^ ^24^ In contrast, Ambroszkiewicz et al. found that patients with bone tumors had a significantly higher percentage of fat and fat body mass after chemotherapy than healthy children. They reported that mean BMI values in adolescent patients with bone tumors after therapy was not different from controls. However, 17% of patients were overweight/obese. Nine percent of these patients were overweight and obese before chemotherapy. Moreover, patients had a significantly higher percentage of fat and fat body mass than healthy children.^25^ The Hematologic Tumor Group had an increase in FM using D_2_O (p=0.011). Our finding regarding the rapid increase in FM during the first months after diagnosis is consistent with those in acute lymphoblastic leukemia (ALL) patients.^3^ Glucocorticoid therapy is the standard for ALL treatment regimens. Such studies showed that weight gain occurs immediately after the start of treatment with dexamethasone, but it is not yet clear whether excess weight persists after treatment.^3^ ^,^ ^9^

Anthropometric measures were able to predict nutritional status in the present study since they were well correlated with D_2_O. Although D_2_O is widely accepted as a useful measure of body composition, especially in research settings, it is seldom available in low-income countries, such as Brazil. However, in these circumstances, arm anthropometry has proven to be valuable as an estimate of nutritional status, correlating with important clinical outcomes in children with cancer.^1^ ^,^ ^16^ Some studies have also revealed strong correlations between triceps skinfold thickness and FM determined by BIA or D_2_O;^26^ ^,^ ^27^ ^,^ ^28^ the same occurring with FFM determined by mid-arm muscle mass circumference and triceps skinfold thickness.^17^ ^,^ ^29^ Other studies with children and adolescents with cancer show that FM can be predicted with reasonable confidence using arm anthropometry. They demonstrated a good correlation between MUAC and DXA.^27^ ^,^ ^30^ These studies suggest that anthropometry may be a useful tool in clinical practice for pediatric cancer patients. This study recommends incorporating arm anthropometry in the routine care of children with cancer. This practice would be especially valuable in low-income countries for being an easy, quick, inexpensive, and reliable method.

A limitation of this study is the inclusion of a relatively small number of children and the heterogeneity of the sample concerning the underlying diagnosis. However, the present study has used one of the most robust methods of body composition and a wider range of techniques than previous investigations to examine cancer children. These findings support the concept that BIA and anthropometry can be used in clinical practice for appropriate measurement of body composition in children with cancer, particularly during the early stages of the disease and its treatment.
